# Do Beliefs About Sexual Orientation Predict Sexual Identity Labeling Among Sexual Minorities?

**DOI:** 10.1007/s10508-022-02465-7

**Published:** 2022-11-16

**Authors:** James S. Morandini, Rachel E. Menzies, Sam G. Moreton, Ilan Dar-Nimrod

**Affiliations:** 1 School of Psychology, The University of Sydney, Sydney, NSW 2006 Australia; 2 School of Psychology, University of Wollongong, Wollongong, Australia

**Keywords:** Sexual orientation, Lesbian, Gay, Bisexual, Sexual orientation beliefs, Queer, Pansexual

## Abstract

Research has found that sexual orientation beliefs predict heterosexuals’ attitudes toward sexual minorities, and important sexual identity outcomes in sexual minority populations. To this point, no studies have systematically examined how sexual orientation beliefs may be associated with sexual identity self-labeling among sexual minority individuals. The present study examined this question in a sample of 1840 same-gender attracted individuals recruited for a cross-sectional online survey. Beliefs in the naturalness and discreteness of sexual orientation categories were highest in gay/lesbian individuals, intermediate in bisexual people, and lower in queer and pansexual individuals. Beliefs in the importance of sexual orientation were highest in gay/lesbian and queer identified individuals and lower in bisexual people. Within-group analysis demonstrated that gay/lesbian individuals who reported more exclusive same-gender attraction reported higher naturalness, discreteness, and importance beliefs than those with less-exclusive same-gender attraction. However, naturalness, discreteness, and importance beliefs were not associated with sexual attraction patterns in bisexual individuals. Finally, among predominately same-gender attracted populations, the adoption of a queer identity (over a gay/lesbian identity) was predicted by lower naturalness and discreteness beliefs, and increased perceived importance in females. Among non-monosexual populations, adoption of a pansexual identity over a bisexual identity was predicted by lower naturalness beliefs in females, but not predicted by sexual orientation beliefs in males. Collectively, these findings suggest that sexual orientation beliefs differ between sexual identity groups and may partly explain the adoption of particular sexual identity labels among contemporary sexual minority populations.

## Introduction

Among laypeople, there exists a range of beliefs about the nature and origins of sexual orientation (Haslam & Levy, 2006; Morandini et al., 2021). Some people believe that sexual orientation is innate, whereas others believe that a person’s sexual orientation is malleable or even chosen (Whisman, 2012). Although some view sexual orientation as existing in two or three discrete categories (gay, straight, or bisexual), others view it as existing on a continuum from exclusively heterosexual to exclusively homosexual with many gradations in between (Kinsey et al., 1948).

These ontological beliefs about sexual orientation have been of interest primarily among social scientists as they appear to be predictive of attitudes toward sexual minority people (Hegarty, 2002). It is important to acknowledge at the outset that the study of sexual orientation beliefs in heterosexual and sexual minority samples has almost exclusively been confined to White Western English-speaking samples, and it is possible that discourses around sexual orientation (as well as their implications) differ in non-Western samples, as well as within minority communities within the West (Furnham & Saito, 2009). At least in Western English-speaking samples, studies have shown that those who viewed homosexuality as innate and immutable were more tolerant of gays/lesbians (Haslam & Levy, 2006; Hegarty, 2002) and those who viewed sexuality as existing in discrete categories were less so (Haslam & Levy, 2006). Only recently have researchers begun investigating how beliefs about sexual orientations may influence elements of attitudes and identity development in sexual minorities individuals, such as internalized homophobia (Morandini et al., 2015), identity uncertainty (Morandini et al., 2017a, 2017b), or identity centrality (Tierney et al., 2021). However, a number of basic, yet important, questions remain regarding sexual orientation beliefs in sexual minority populations. Key among these are whether sexual orientation beliefs: (1) differ across diverse sexual identity groups, and (2) influence the adoption of sexual identity label. The latter possibility may be particularly relevant in explaining why people with seemingly similar sexual attraction patterns might adopt different sexual identity labels, particularly non-traditional sexual identity labels such as pansexual or queer. Pansexual is a sexual identity that typically denotes attraction to people regardless of their gender identity or birth-assigned gender (Rice, 2015). It should be noted, however, that many individuals who identify as bisexual reject the notion that a bisexual label excludes attraction to transgender/non-binary individuals or imply an ideological commitment to the gender binary (Oritz, 2016). Queer, which originally was a pejorative term for homosexual men (Chauncey, 1994), is now a common sexual minority identity label. Queer is sometimes preferred as a sexual identity label as it defies normative categories of homosexual vs. bisexual vs. heterosexual, which may be viewed as artificial, essentialist, and/or oppressive (Horner, 2007). Like pansexual, it may also be preferred because it is seen as more inclusive of attractions to transgender folk and those outside of the sex/gender binary. Again, it should be noted that “queer” and “pansexual” identities may be constructed in distinct ways across culture, and as such our discussion of the intersection of these sexual identities and sexual orientation beliefs, is necessarily limited to majority white, Western English-speaking samples.

Morandini et al. (2017b) demonstrated that bisexual and pansexual individuals do not differ when asked to report their sexual attraction to same- and opposite-sex partners (i.e., their sexual attraction patterns), despite selecting different identity labels to represent their sexual orientation. Moreover, a good proportion of queer identified individuals had non-monosexual sexual attractions (consistent with those of bisexual- and pansexual-identified individuals) while others had attractions consistent with gay/lesbian individuals. Thus, while sexual identity labels are typically related to sexual attraction patterns, these constructs are clearly not identical.

The distinction between these two constructs is essential to the present inquiry, given we are interested in factors other than sexual attraction pattern, that may influence one’s sexual identity self-labeling. Sexual attraction pattern, as measured by the Kinsey Scale, relates to the degree to which one experiences erotic thoughts and feelings toward males, females, or both (Bailey, 2009). Sexual identity, on the other hand, can refer to a range of psychological phenomena, including (but not limited to), sexual identity self-label, degree of affiliation with a particular sexual minority community, and degree of integration of one’s sexual orientation into one’s private and public persona. Sexual identity (unlike sexual attraction pattern) is necessarily socially and historically contingent—and can be shaped by a range of cultural and idiosyncratic factors. In a trivial sense this is obvious, i.e., the specific labels used to refer to non-monosexual people differ between cultures and across historical periods—e.g., “pansexual” in its current meaning seems to have emerged among Western millennials and Gen Z’s (cohorts of individuals born between 1981–1996 and 1997–2012 respectively) in online forums such as Tumblr™ in recent years (Gonel, 2013). Perhaps less obvious, are the ways in which distinct attributions regarding the significance and meaning of one’s sexual attraction pattern (perhaps due to social, cultural, or idiosyncratic factors) can lead to meaningfully distinct psychological identities and experiences. For instance, some heterosexual identified women may experience occasional sexual fantasies involving other women but deem such experiences as universal to women, and not indicative that they are bisexual. Others, with identical attraction patterns, may believe these feeling signal they could be distinct from heterosexual women, might be compelled to explore these feelings further, might affiliate and develop a sense of community and belonging with queer women, and eventually come to see themselves as bisexual. In this way, sexual identity development, for some individuals, can be seen as akin to the development of other complex social–psychological identities (e.g., vocational, political, or religious identities) (Kitzinger, 1987).

Relevant to this point, differences in how non-monosexual women construct their sexual identity—which are potentially influenced by their beliefs about sexual orientation—can impact identity related stressors and mental health. Research suggests that while bisexual- and pansexual-identified women may not demonstrate significant differences in their sexual attraction patterns, the psychological antecedents that predict their wellbeing (and mental health) nevertheless differ (e.g., stigma consciousness predicts lower wellbeing among pansexual women more strongly than among bisexual women—Morandini et al., 2021). As such, sexual orientation beliefs may have influences on both how one conceives of their sexual identity, and in turn experiences of minority stress and wellbeing.

### Sexual Orientation Beliefs Among Sexual Minorities

Whereas beliefs about sexual orientation span a wide variety of areas, they may be focused on origin (e.g., innate), their nature (e.g., malleable), and consequences (e.g., superior status), among other, research on those beliefs have focused primarily on a few dimensions. In the present study, we maintain the focus on some of these specific beliefs relating to immutability, discreteness, importance, and entitativity. These beliefs map onto facets most widely studied within the essentialist literature (Haslam et al., 2002)—and are derived from Arseneau et al.’s (2013) Sexual Orientation Beliefs (SOB)’s scale. To our knowledge, this is the only existing measure of ontological beliefs about sexual orientation that has been validated in a sexual minority sample.

No published research to date has systematically compared those sexual orientation beliefs among gay, lesbian, or bisexual sexual identity groups, let alone, among those with non-traditional sexual identities. However, existing literature suggests that some differences between gay/lesbian and bisexual populations are to be expected. In the domain of immutability, Herek et al. (2009) found that 55% of bisexual women reported some degree of choice in their sexual orientation, compared to just 30% of lesbian women. This may be because bisexuals actually experience more malleability in their sexual feelings over time, compared with monosexuals (Diamond, 2008b). Alternatively, it may be because immutability beliefs are strategically less useful in defending one’s bisexuality from pressure to conform to heteronormativity—as, unlike homosexual individuals, a bisexual person could hypothetically have a successful heterosexual relationship if they chose to (Hubbard & de Visser, 2015). Herek et al. also found that among men the reported degree of choice in their sexual orientation also differed (13% vs 41% for gay vs bisexual men respectively), indicating not only that the self-reported labels are important, but also that the endorsement of such essentialist beliefs vary considerably by gender, and thus, there is little justification in ignoring gender for such analyses. Morgenroth et al. (2021) found other aspects of sexual orientation essentialism to be lower among bisexual individuals than gay/lesbian individuals. Specifically, they showed that bisexual people see sexual orientation as less natural and disecrete than their gay/lesbian counterparts. The lower naturalness perception among bisexual individuals predicted reduced sense of belonging to the LGBT + community.

In addition to beliefs pertaining to the naturalness and discreteness of sexual orientation, two other types of sexual orientation beliefs that have received less attention are *importance* (i.e., viewing sexual orientation as being socially and personally central to a person’s identity) and *entitativity* (i.e., viewing sexual orientation groups as uniform, consisting of homogenous individuals, and identity labels as being informative of an individual’s character). The former might be expected to differ between monosexuals and non-monosexuals, as gay/lesbian sexual identities tend to be more visible and socially salient than bisexual identities, as bisexuals may be currently in heterosexual relationships (Tabatabai, 2015). As a result, gay/lesbian individuals are likely to find sexual orientation of more importance, on average, than bisexuals. The latter belief, entitativity, due to its conceptual similarity to stereotype endorsement (Bastian & Haslam, 2006) may have interesting correlates in sexual minorities (e.g., greater internalized stigma). However, it is less obvious how entitativity might differ between members of sexual identity groups themselves. In the only study that assessed differences between bisexual and gay/lesbian individuals (Morgenroth et al., 2021), no significant differences on this dimension were found between these populations.

A recent study by Tierney et al. (2021) used latent profile analysis to identify sexual orientation belief profiles in a sample of 416 sexual minority individuals examining how these profiles were associated with sexual identity development outcomes. A three-profile solution was identified, with profiles labeled “naturalness-only” (i.e., high naturalness, low discreteness/importance/entitativity), “multidimensional essentialism” (i.e., high on all of these essentialist beliefs), and “high discreteness, entitativity, and importance” (i.e., high on all essentialist beliefs except naturalness). Monosexuality (gay/lesbian identity) predicted membership of the naturalness-only profile over the multidimensional essentialism profile, whereas non-monosexuality predicted membership the multidimensional essentialism profile over the naturalness-only profile. However, it is notable that this study did not examine how participant gender may interact with sexual orientation in predicting sexual orientation beliefs, predictors of within sexual identity group variability in beliefs, nor how sexual orientation beliefs may differ among queer or pansexual populations. In fact, no past research has compared mean differences in sexual orientation belief factors between sexual identity groups.

Recent literature has found that there is more within-group variability among gay, lesbian, and bisexual groups than previously thought (Vrangalova & Savin-Williams, 2012). Whereas some gay and lesbian individuals are exclusively same-gender attracted, others experience non-exclusive same-gender attraction. While “gay” or “lesbian” identity labels might be the most easily communicated social identity available for such individuals, it is possible that their non-categorical patterns of attraction may lead them to view sexual orientation in a less immutable or discrete manner. Such a shift in beliefs would be in line with recent experimental evidence, which demonstrated that exposure to less discrete views of sexuality can actually increase endorsement of non-exclusive same-gender attractions (Morandini et al., 2021). Thus, it is reasonable to expect an association between experiencing gender-non-exclusive attraction among gay/lesbian individuals and lower beliefs about the discreteness of sexual orientation.

A similar phenomenon may exist among bisexual individuals. Those who experience relatively equal attraction to males and females (i.e., who rate their attraction as the midpoint of the Kinsey scale) may feel more comfortable with a categorical understanding of sexual orientation generally, and bisexuality specifically, than those who are gay- or straight leaning (whose sexual attraction patterns are better captured by a more continuous conceptualization of sexual orientation). As such, bisexual individuals may hold more categorical views of sexuality as they are closer to the prototypical of the category. Therefore, it may be expected that there may be a U-shaped relationship between Kinsey scores and endorsement of categorical views of sexuality, with bisexual identifying individuals at either end of the Kinsey scale reporting lower endorsement of these beliefs than those in the middle.

### Sexual Orientation Beliefs and Non-Traditional Sexual Identity labels

An increasing proportion of same-gender attracted individuals are reporting non-traditional sexual identities; that is, identities other than lesbian, gay, bisexual, or straight (Horner, 2007; Savin-Williams, 2009; Watson et al., 2019). This trend has perhaps been most clearly documented among young people, with around a quarter of contemporary same-gender attracted youth identifying with a non-traditional label (Porta et al., 2020; Watson et al., 2019). While various non-traditional labels exist, the two most common are “pansexual” and “queer” (Mitchell et al., 2014; Watson et al., 2019).

There has been growing interest in exploring differences between those who identify with traditional sexual identity labels (gay/lesbian/bisexual) and those who identify as pansexual or queer. For example, a thematic analysis of qualitative responses demonstrated that bisexual- and queer-identifying individuals are significantly more likely to report a distinct preference for one gender (e.g., explicitly preferring women; Galupo et al., 2017) compared to pansexual individuals. Further, pansexual individuals are more likely to describe their attraction as transcending gender or sex, compared to those adopting bisexual or queer labels. Pansexual and queer individuals are also less likely than bisexual individuals to describe their sexual identity in terms of an explicit gender binary (Galupo et al., 2017). In a similar vein, recent studies demonstrate that individuals who identify as bisexual are older and more politically conservative than those identifying as pansexual (Greaves et al., 2019).

However, other studies reveal mixed findings on the differences between those adopting non-traditional labels. Notably, one study has shown that individuals who identify as pansexual do not differ from those who identify as bisexual on indices such as sexual attraction, romantic attraction, sexual behavior, and partner gender (Morandini et al., 2017b). In contrast, the majority of individuals identifying as queer demonstrate predominately homosexual patterns of sexual attraction. Such findings suggest that differences among these labels may be explained more by beliefs (e.g., beliefs about sexuality, gender or politics more generally; Greaves et al., 2019) than by actual sexual preferences.

### The Present Study

There are several gaps in the existing literature that the present study aimed to fill. To this point, no studies have systematically examined differences in sexual orientation beliefs across a broad range of sexual minority cisgender men and women. Moreover, research pertaining to sexual orientation beliefs has largely focused on samples using traditional identity labels, limiting conclusions about beliefs among those selecting non-traditional identity labels. Thus, while we know that biological determinist views of sexual orientation currently predominate among gay and lesbian individuals (Morandini et al., 2015, 2017a, 2017b), we know little about how pansexual or queer populations conceive of sexuality.

Further, it is possible that differences in beliefs about sexual orientation are explained by variations in sexual attraction that are not well captured by common identity labels. As mentioned, growing research finds substantial within-group variability in attraction patterns among bisexual, gay, and lesbian individuals (Feinstein et al., 2015), with evidence of intermediary categories of sexual orientations (e.g., “mostly straight” or “mostly gay/lesbian”; Morandini et al., 2019; Vrangalova & Savin-Williams, 2012). Individuals whose pattern of attraction falls in these intermediate ranges may be more likely to endorse beliefs that reflect this (e.g., reduced beliefs in naturalness, discreteness, importance). Given this within-group variability, one prevalent limitation of previous studies is the reliance on identity labels, while ignoring sexual attraction patterns.

Perhaps most speculative, is the possibility that sexual orientation beliefs, not sexual feelings per se, are what most differentiate some decisions about sexual identity self-labeling. For instance, why do some individuals who score a five on the Kinsey scale (mostly same-sex attracted) identify as gay, whereas others identify as bisexual, or even queer? Is it that part of what differentiates such individuals may be whether they conceive of sexuality as categorical versus continuous, or fluid versus fixed? If so, sexual identity labeling, in some instances, may be as much about how one conceptualizes sexuality as how one experiences it.

In the present study, we aimed to draw upon a large online sample of same-gender attracted cisgender individuals to examine how beliefs differ across and within diverse sexual identity groups. We hypothesized that:

1. Gay- and lesbian-identified individuals would endorse greater sexual orientation essentialism (i.e., higher naturalness, discreteness, and importance) than non-monosexuals (queer-, bisexual-, and pansexual-identified individuals).
2. Among non-monosexuals, those adopting non-traditional sexual identity labels (i.e., pansexual and queer) would report lower naturalness and discreteness beliefs than bisexual individuals.
3. Gay, lesbian, and bisexual individuals, with non-prototypical attractions (e.g., gay men with non-exclusive same-gender attraction or bisexual women who are lesbian/straight leaning) would endorse lower naturalness and discreteness beliefs than individuals with prototypical attractions.
4. Controlling for sexual attraction patterns and age, lower naturalness and discreteness beliefs would predict greater likelihood of adopting a queer identity than a gay/lesbian identity. We chose this comparison as evidence suggests that gay/lesbian versus queer populations demonstrate similar same-sex sexual attraction patterns, and thus perhaps distinct beliefs about sexual orientation may contribute to divergent sexual identity labeling (Morandini et al., 2017b).
5. Controlling for sexual attraction patterns and age, lower naturalness and discreteness beliefs would predict greater likelihood of adopting a pansexual identity than a bisexual identity—given evidence that pansexual and bisexual populations tend to share a sexual attraction pattern as measured by the Kinsey scale—suggesting distinct beliefs about sexuality may contribute to distinct sexual identity labeling in these two populations (Morandini et al., 2017b).
6. Finally, controlling for sexual attraction patterns and age, lower naturalness and discreteness beliefs would predict greater likelihood of adopting a bisexual identity than a gay/lesbian identity. We included this comparison to examine whether sexual orientation beliefs may be predictive of adoption of distinct normative sexual identity labels (e.g., gay/lesbian versus bisexual) in addition to their contribution to predicting the choice of a non-traditional label over a traditional label among individuals likely to demonstrate similar sexual attraction patterns.

We also undertook exploratory analysis to examine how entitativity may differ across sexual identity and gender, given its failure to differ between sexual minority groups in a past study (Morgenroth, Kirby, Gee, & Ovett, 2021).

## Method

### Participants

The participants in the present study were recruited as part of a large study investigating psychological wellbeing among same-gender attracted (SGA) individuals (see further Hunt et al., 2020; Morandini et al., 2017a, 2017b). Recruitment for the large study occurred through targeted social media advertisements and snowball sampling. Advertisements for the study were posted in various LGBTIQ social media groups, and a paid advertisement was placed in a popular national LGBTIQ website. E-mail invitations for the study were also distributed to mailing lists of local LGBTIQ organizations. The study was approved by an institutional Ethics Committee. The present study utilized a subset of data from participants who had completed the sexual orientation beliefs scale, Kinsey Scale, and reported a sexual identity label (not including “other” that was deemed to heterogeneous to be a meaningful group for comparison) and a cisgender identity.

There is substantial evidence that transgender/non-binary individuals conceive of and construct their sexual identities in ways that are distinct from that in cisgender individuals (Dickey et al., 2012; Galupo et al., 2016), requiring that cisgender and non-cisgender participants sexual orientation beliefs be examined separately. There were too few transgender/non-binary respondents in each sexual identity group in our sample for such comparisons to be made. For this reason, we focused this study on sexual orientation beliefs among cisgender sexual minorities—and acknowledge the need for future research examining sexual orientation beliefs in non-cisgender samples. Thus, when we refer to men and women in our sample henceforth, we actually refer to cismen and ciswomen specifically.

The final sample included 950 individuals; (51.4%) men (Gay = 823 [86.6%]; Bisexual = 89 [9.4%]; Queer = 27 [2.8%]; and Pansexual = 11 [1.2%]), and 898 (48.6%) women (Lesbian = 483 [53.8]; Bisexual = 266 [29.6]; Queer = 84 [9.4]; Pansexual = 65 [7.2%]). Participants ranged in age from 18 to 70 years (*M* = 30.04, *SD* = 11.32). Self-reported ethnicity was comprised of 83.2% White, 3.8% Asian, 5.4% Mixed Race, 0.9% Middle Eastern, 1% Latin American, 0.8% Aboriginal, 0.2% Pacific Islander, and 0.2% African. With regard to highest level of education, 20.2% held a postgraduate degree, 36.3% held a bachelor’s degree, 39.4% had completed secondary school, and 4.1% had not finished secondary school.

### Procedure

Participants who responded to online advertisements were directed to the online participant information statement and consent form. The following online survey included demographic questionnaires and the measures reported below. Additional measures were also completed, and are reported elsewhere (Morandini et al., 2015a, 2015b, 2017a, 2017b). The survey took approximately 30 min to complete.

### Measures

#### Demographics

Participants were asked to report demographics including their age, sex/gender identity, ethnicity, and education level.

#### Sexual Identity

To assess sexual identity, participants were asked: “What identity label do you use to describe your sexual orientation?”. The response options were “Gay/Lesbian,” “Bisexual,” “Queer,” “Pansexual,” and “Other” (for this latter option, participants were prompted to write in their sexual identity). There were no definitions regarding what these sexual identity labels referred to—rather we were assessing participants self-identification. Participant could only select one sexual identity label.

#### Kinsey Scale (Kinsey et al., 1948)

To assess sexual attraction pattern, a Kinsey type scale assessing present attraction was deployed: “To whom are you sexually attracted?” Responses were registered on a 7-point continuum, (1) exclusively opposite-sex attracted, (2) mostly opposite-sex attracted, (3) somewhat more opposite-sex attracted, (4) equal same and opposite-sex attracted, (5) somewhat more same-sex attracted, (6) mostly same-sex attracted, and (7) exclusively same-sex attracted. The Kinsey Scale is a widely deployed in sexuality research and demonstrates good convergent and predictive validity with sexual identity self-labeling and psychophysiological sexual arousal pattern, particularly in men (Bailey, 2009). That said, critiques have been made of face validity of items, particularly when applied to gender diverse samples (Galupo et al., 2017).

#### Sexual Orientation Beliefs Scale (SOBS; Arseneau et al., 2013

A 35-item self-report measure of beliefs about sexual orientation was utilized in this study. The SOBS assesses the level of perceived *Naturalness* (e.g., “biology is the main basis of an individual’s sexual orientation”; 12 items), *Discreteness* (e.g., “Sexual orientation exists in categories with clear boundaries: A person is either homosexual, bisexual, or heterosexual”; four items), *Entitativity* (e.g., “People who share the same sexual orientation pursue common goals”; 10 items), and *Importance* (e.g., “If you don’t know a person’s sexual orientation you can’t really say you know that person”; seven items) of sexual orientation.^1^ Each item was rated on a 5-point scale ranging from a 1 (“Strongly disagree”) to 5 (“Strongly agree”). The SOBS has been shown to have good internal reliability and test–retest reliability (Arseneau et al., 2013). In the current study, the internal consistencies of these subscales were generally acceptable. See Table 1 for internal consistency of sexual orientation beliefs as a function of gender and sexual orientation. A validation study of the SOBS found that it demonstrated configural invariance across a diverse LGBTIQ sample and a heterosexual sample—indicating that the four-factor structure was invariant across groups and is appropriate for use in both populations. That being said, more stringent tests of measurement invariance, including within subgroups of LGBTIQ folk (based on sexual orientation and gender identity), are yet to be established. One existing study by Morandini et al. (2017a, 2017b) demonstrated a violation of configural invariance for the naturalness subscale of the SOBS between cisgender lesbian versus bisexual women—suggesting future work needs to assess measurement invariance of the scale as applied to other LGBTIQ subgroups. Although a growing body of work has shown relationships between SOB subscales in sexual minority populations (e.g., internalized homophobia [Morandini et al., 2015]) and heterosexual populations (e.g., anti-gay attitudes [Grzanka et al., 2016]), more data on the predictive, convergent, and discriminative validity of the SOBS are needed (Arseneau et al., 2013).

**Table 1:** Internal reliability of sexual orientation beliefs subscales by sexual identity and gender

	Naturalness (α)	Discreteness* (α)	Entitativity (α)	Importance (α)
*Lesbian/Gay*				
Women Men	.721 .742	.770 .798	.808 .809	.744 .734
*Bisexual*				
Women Men	.699 .795	.659 .623	.762 .838	.718 .738
*Queer*				
Women Men	.733 .867	.803 .768	.775 .764	.721 .556
*Pansexual*				
Women Men	.532 .720	.646 .601	.747 .851	.661 .782

^*^Two items (17 and 18) were dropped from the discreteness subscale (for all participants) to ensure that Cronbach alpha was above .60 in pansexual males or females

### Statistical Analyses

The data were analyzed using SPSS version 26. A series of univariate analyses of variance (ANOVA) were conducted to explore the hypotheses regarding gender and sexual identity. Where omnibus tests were significant, simple effects analyses were conducted to examine differences between groups. A series of regression analyses helped determine whether sexual attraction patterns predicted within-group sexual orientation beliefs among individuals adopting traditional labels. In each regression, participants’ mean-centered scores on the Kinsey scale were entered along with gender (0 = man, 1 = woman) and a gender by Kinsey score interaction term. We also included a quadratic term to test for the hypothesized quadratic relationship for bisexuals whereby beliefs might shift the further away bisexuals were from the midpoint of the Kinsey scale. For the purpose of consistency, we also included a quadratic term for the analyses for gay/lesbian individuals, although there were no hypothesized quadratic effects. Neither queer nor pansexual groups were analyzed in this manner due to insufficient sample sizes.

Finally, a series of simultaneous binomial logistic regressions examined whether sexual orientation belief factors (naturalness, discreteness, entitativity, and importance) predicted identification with certain sexual identities, above and beyond sexual attraction pattern and age. We controlled for sexual attraction pattern for obvious reasons (i.e., it is perhaps the primary construct informing sexual identity), but also age, given that age was associated with adoption non-monosexual identities as well as sexual orientation beliefs in our data. Sexual identity was coded into three pairs of contrasts corresponding to our key hypotheses. These were: gay = 0 versus bisexual = 1, gay = 0 versus queer = 1, and bisexual = 0 versus pansexual = 1. Binary logistic regressions were run in two steps. In the first step were the control variables sexual attraction and age. In the second step were the four sexual orientation belief factors. Separate analyses were conducted for men and women. The *p-*criterion was set at 0.05.

## Results

### Data Preparation

First, we assessed the data for missingness. From 2,445 who accessed the survey, 2,220 attempted all demographic questions of interest. Of this group, *n* = 129 were non-cisgender and *n* = 34 participants reported a sexual identity other than gay, lesbian, bisexual, pansexual, or queer (selecting the “Other—please specify” option; see Morandini et al., 2017a, 2017b for a breakdown of sexual identities written in the adjoining open-text box). These participants were removed from the sample. N = 1847 participants attempted the Sexual Orientation Beliefs Scale [SOBS]. Missing data analysis indicated that less than 1.5% of data were missing for any one item of the SOB scale among those who had attempted the SOBS. Pair-wise deletion was employed in analyses given that missing rates < 5% are inconsequential and imputation approaches not shown to be superior in such cases (Kang, 2013).

### Do Sexual Orientation Beliefs Differ by Gender and Sexual Identity?

**Fig. 1:**
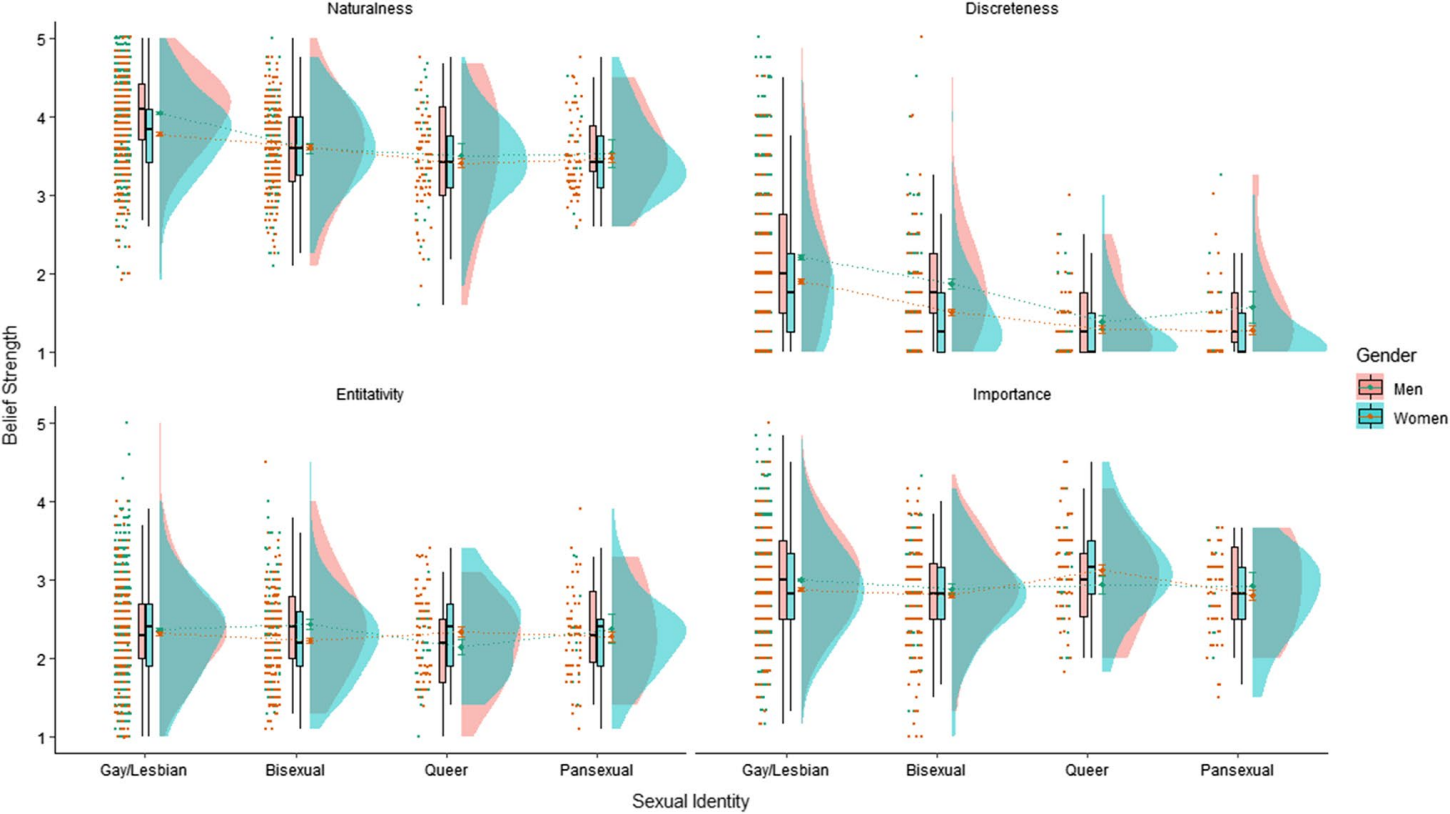
Sexual orientation beliefs across sexual identity groups

Descriptive statistics and visualization of sexual orientation beliefs across gender and sexual identity can be seen in Table 2 and Fig. 1. Figure 1 utilizes raincloud plots (Allen et al., 2019), with each jittered dot representing an individual’s score. The smoothed half-violin plots represent the smoothed frequency distribution. The box and whisker plots represent the median (the line within the box), interquartile range (edge of the boxes), and 1.5 times the interquartile range above and below (the whiskers). Mean and standard error are represented by the dot and confidence bands located between the box and half-violin components of the figure.

**Table 2:** Descriptive statistics and results from comparisons of sexual orientation beliefs within and between gender and sexual identity

Sexual orientation beliefs	Gay/lesbian M (SD)	Bisexual M (SD)	Queer M (SD)	Pansexual M (SD)
*Naturalness*				
Women	3.77 (0.53)	3.61 (0.51)	3.40 (0.53)	3.47 (0.48)
Men	4.04 (0.51)	3.59 (0.62)	3.50 (0.80)	3.53 (0.58)
Total	3.94 (0.53)	3.61 (0.51)	3.43 (0.60)	3.48 (0.49)
*Discreteness*				
Women	1.90 (0.72)	1.50 (0.56)	1.29 (0.43)	1.27 (0.42)
Men	2.20 (0.83)	1.87 (0.67)	1.38 (0.47)	1.57 (0.68)
Total	2.09 (0.80)	1.59 (0.61)	1.31 (0.44)	1.32 (0.47)
*Entitativity*				
Women	2.32 (0.57)	2.22 (0.51)	2.34 (0.51)	2.28 (0.52)
Men	2.37 (0.59)	2.44 (0.61)	2.15 (0.51)	2.38 (0.63)
Total	2.35 (0.59)	2.28 (0.55)	2.29 (0.52)	2.30 (0.54)
*Importance*				
Women	2.88 (0.60)	2.81 (0.57)	3.13 (0.55)	2.80 (0.51)
Men	3.00 (0.61)	2.88 (0.58)	2.94 (0.58)	2.92 (0.57)
Total	2.95 (0.61)	2.83 (0.57)	3.08 (0.56)	2.82 (0.52)

### Naturalness (Biological/Immutability Beliefs)

Beliefs in the biological basis and immutability of sexual orientation were compared across gender and sexual identity with a main effect of sexual identity [*F*(3,1840) = 45.61, *p* < 0.001, η_p_^2^ = 0.07] and a gender by sexual identity interaction observed [*F*(3,1840) = 6.39, *p* = 0.001, *η*_p_^2^ = 0.01; see Table 2 for descriptives]. There was no main effect of gender [*F*(1,1840) = 3.32, *p* = 0.07, η_p_^2^ = 0.002). Analyses of simple effects revealed that gay men perceived sexual orientation as more biologically based and immutable than bisexual, queer, or pansexual men (*p*’s < 0.001), who were not significantly different in regards to these beliefs (*p*’s > 0.10). Among women, those identifying as lesbian viewed sexual orientation as more biological/immutable/fixed than bisexual (*p* = 0.001) and queer/pansexual-identified (*p*’s < 0.001) women. Bisexual women viewed sexual orientation as more biological/immutable than those identified as queer (*p* = 0.04) or pansexual (*p* = 0.004), who in turn did not significantly differ from each other (*p* = 0.47).

### Discreteness Beliefs

An ANOVA of the beliefs in the discreteness of sexual orientation categories by gender identity revealed a main effect of gender [F(1,1828) = 12.08, *p* = 0.001, η_p_^2^ = 0.01; see Table 2 for descriptives] and sexual identity [*F*(3,1828) = 45.53, *p* < 0.001, η_p_^2^ = 0.07], but no significant interaction *F*(3,1828) = 0.73, *p* = 0.54, η_p_^2^ = 0.001]. Overall, women reported sexual orientation as less discrete/categorical than men (*p* < 0.001). Bivariate comparisons for sexual identity revealed that those who identified as gay/lesbian viewed sexual orientation categories as more discrete/categorical than those who identified as bisexual, queer, or pansexual (*p*’s < 0.001). In turn, bisexual individuals perceived sexual orientation as more discrete than queer (*p* < 0.001) or pansexual (*p* = 0.04) individuals, who did not differ from one another (*p* = 0.55).

### Entitativity Beliefs

An ANOVA of entitativity beliefs by gender identity revealed a significant interaction between sexual identity and gender [*F*(3,1822) = 3.01, *p* = 0.03, η_p_^2^ = 0.01], but no main effects of either sexual identity [*F*(3,1822) = 0.80, *p* = 0.49, η_p_^2^ = 0.001] nor gender [*F*(1,1822) = 0.52, *p* = 0.47, η_p_^2^ < 0.001]. Bivariate comparisons revealed that queer men had lower entitativity beliefs than gay (*p* = 0.046) and bisexual men (*p* = 0.02). No other bivariate comparisons among men were significant (*p*’s > 0.05). Lesbian women had higher entitativity beliefs than bisexual women (*p* = 0.03). No other bivariate comparisons among women were significant (*p*’s > 0.05).

### Importance Beliefs

An ANOVA of importance beliefs by gender and sexual identity revealed a significant main effect of sexual identity [*F*(3, 1816) = 2.29, *p* = 0.034, η_p_^2^ = 0.01]. There was no significant main effect of gender [*F*(1, 1816) = 0.28, *p* = 0.60, η_p_^2^ < 0.001], nor interaction between gender and sexual identity [*F*(3, 1816) = 1.67, *p* = 0.17, η_p_^2^ = 0.003]. Bivariate comparisons revealed that bisexual individuals had lower importance beliefs than gays/lesbian (*p* = 0.02) or queer (*p* = 0.01) persons. No other bivariate comparisons were significant (*p*’s > 0.05).

### Do Differences in Sexual Attraction Pattern Predict Sexual Orientation Beliefs Within Sexual Identity Groups?

A series of linear regressions tested whether sexual orientation beliefs were significantly predicted by sexual attraction patterns. In each regression, participants’ mean-centered scores on the Kinsey scale were entered along with gender (0 = men, 1 = women), a gender by Kinsey score interaction term, and the above-mentioned quadratic term that captures the distance from Kinsey value that is considered prototypical for the individuals in the sexual orientation category (e.g., the Kinsey midpoint for bisexual-identified individuals).

### Naturalness

#### Gays/Lesbian Individuals

**Fig. 2:**
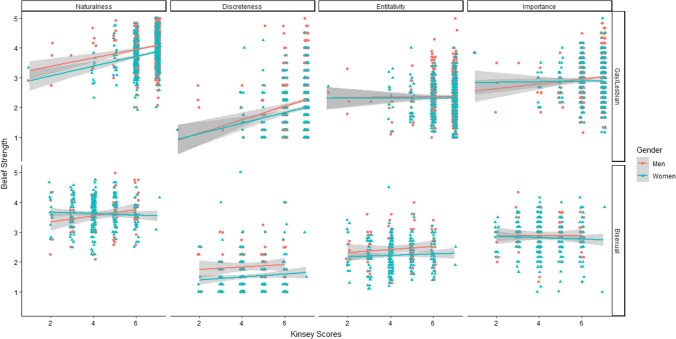
Linear relationships between sexual attraction patterns and sexual orientation beliefs in gay/lesbian and bisexual individuals

As seen in Fig. 2, naturalness beliefs were positively predicted by Kinsey scores (*β* = 0.20, *t* = 5.30, *p* =  < 0.001), indicating that people lower in other-gender attraction held stronger naturalness beliefs. Gender negatively predicted naturalness beliefs (*β* = −0.21, *t* = −5.81, *p* =  < 0.001), indicating gay men overall had stronger naturalness beliefs than lesbian women. There was no significant interaction between sexual attraction and gender (*β* = 0.02, *t* = 0.41, *p* = 0.68), nor a significant quadratic effect (*β* = 0.04, *t* = 1.32, *p* = 0.19).

#### Bisexual Individuals

There was a significant gender by sexual attraction interaction (*β* = −0.28, *t* = 2.22, *p* = 0.03). There were no significant main effects of gender (*β* = -0.14, *t* = −1.57, *p* = 0.12) or sexual attraction (*β* = 0.15, *t* = 0.94, *p* = 0.35). The quadratic term was also nonsignificant (*β* = −0.06, *t* = -0.41, *p* = 0.68). We conducted follow-up simple slope analyses by analyzing men and women in separate regressions with naturalness beliefs regressed on the sexual attraction linear and quadratic terms. There were no linear effects of sexual attraction for either men (*β* = 0.29, *t* = 0.93, *p* = 0.35) or women (*β* = −0.16, *t* = −0.99, *p* = 0.32). Thus, while the interaction suggested a difference in the relationship between naturalness beliefs and sexual attraction patterns in bisexual men vs bisexual women, the relationship was not significantly different from 0 in either bisexual men or bisexual women. There were no quadratic effects for either men (*β* = 0.10, *t* = 0.31, *p* = 0.76) or women (*β* = −0.11, *t* = −0.71, *p* = 0.48).

### Discreteness

#### Gays/Lesbian Individuals

**Fig. 3:**
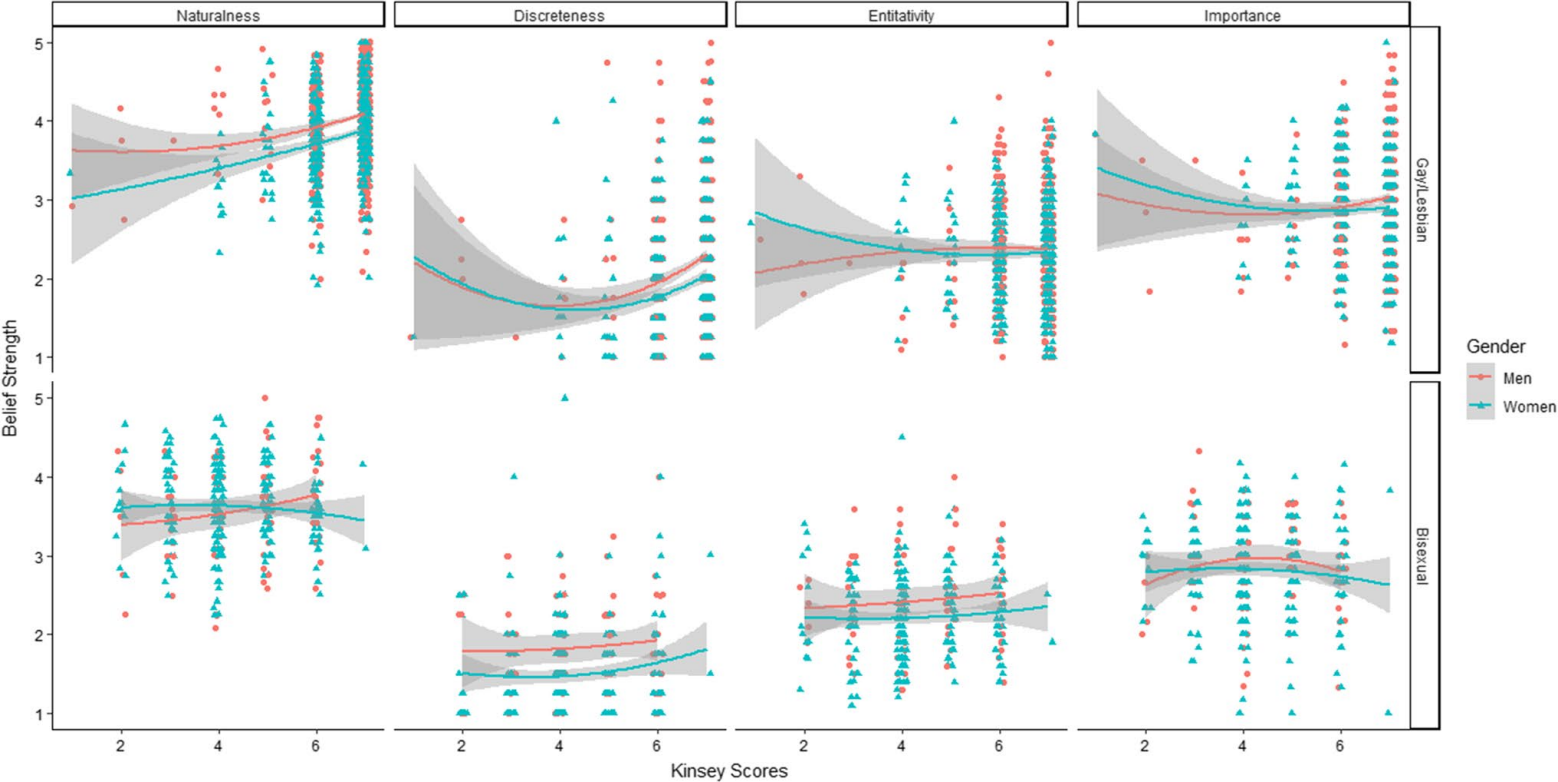
Quadratic relationships between sexual attraction patterns and sexual orientation beliefs in gay/lesbian and bisexual individuals

A significant effect of sexual attraction emerged, such that higher scores on the Kinsey scale predicted higher discreteness beliefs (*β* = 0.24, *t* = 6.25, *p* =  < 0.001). Additionally, a significant effect of gender indicated that gay men held stronger discreteness beliefs than lesbian women (*β* = −0.11, *t* = 3.00, *p* = 0.003). There was also a significant quadratic effect of sexual attraction, such that the relationship between sexual attraction and discreteness beliefs became stronger with higher scores on the Kinsey scale (*β* = 0.11, *t* = 3.83, *p* < . 001) (see Fig. 3). There was no significant interaction between gender and sexual attraction (*β* = −0.05, *t* = −1.13, *p* = 0.26).

#### Bisexual Individuals

A significant effect of gender emerged, such that bisexual men reported stronger discreteness beliefs than bisexual women (*β* = −0.23, *t* = −2.55, *p* = 0.01). There was no significant effect of sexual attraction (*β* = 0.22, *t* = 1.36, *p* = 0.18) nor significant interaction between gender and sexual attraction (*β* = 0.02, *t* = 0.14, *p* = 0.89). There was also no significant quadratic effect of sexual attraction (*β* = 0.15, *t* = 1.11, *p* = 0.27).

### Entitativity

#### Gay/Lesbian Individuals

There were no significant effect of sexual attraction (*β* = 0.01, *t* = 0.31, *p* = 0.75), gender (*β* = −−0.04, *t* = −1.04, *p* = 0.30) nor interaction between them (*β* = −0.01, *t* = −0.14, *p* = 0.89). There was also no significant quadratic effect of sexual attraction (*β* = 0.002, *t* = 0.07, *p* = 0.94).

#### Bisexual Individuals

Bisexual men held stronger entitativity beliefs than bisexual women (*β* = −0.20, *t* = 2.20, *p* = 0.03). There was no significant effect of sexual attraction (*β* = 0.17, *t* = 1.01, *p* = 0.31), nor interaction between sexual attraction and gender (*β* = −0.06, *t* = −0.48, *p* = 0.63). There was also no significant quadratic effect of sexual attraction (*β* = 0.07, *t* = 0.47, *p* = 0.64).

### Importance

#### Gay/Lesbian Individuals

A significant effect of sexual attraction emerged, such that higher scores on the Kinsey scale predicted stronger importance beliefs (*β* = 0.11, *t* = 2.87, *p* = 0.004). There was also a significant quadratic effect, such that this relationship was stronger at the higher end of the Kinsey scale (*β* = 0.06, *t* = 2.04, *p* = 0.04). There were neither significant effect of gender (*β* = −0.04, *t* = 1.01, *p* = 0.32) nor interaction between gender and sexual attraction (*β* = −0.07, *t* = 1.57, *p* = 0.12).

#### Bisexual Individuals

There were no significant effects of sexual attraction (*β* = −0.17, *t* = 1.02, *p* = 0.31), gender (*β* = −0.10, *t* = −1.13, *p* = 0.26) nor the interaction between them (*β* = −0.06, *t* = −0.49, *p* = 0.62). There was also no significant quadratic effect of sexual attraction (*β* = −0.19, *t* = −1.36, *p* = 0.17).

#### Can Sexual Orientation Beliefs Predict Sexual Identity?

As mentioned above, binomial regressions were utilized to assess whether sexual orientation beliefs can predict sexual identity, after controlling for sexual attraction patterns and age. We conducted a series of head-to-head comparisons, separately in women (see Table 3) and men (see Table 4).

**Table 3:** Sexual orientation beliefs as predictors of sexual identity label (women)

	Lesbian versus bisexual			Lesbian versus queer			Bisexual versus pansexual		
Variable	*β*	95% CI	Exp(β)	*β*	95% CI Exp(β)		*β*	95% CI Exp(*β*)	
*Model 1*									
Step χ^2^(1)	284.538*			36.821*			8.058*		
Nagelkerke *R*^*2*^	.490			.042			.073		
Age	−.040**	[.978,1.019]	.961	−.034*	[1.219, 4.014]	.967	−.121*	[1.598, 5.901]	.886
Sexual attraction	−1.083***	[ .059, .518]	.338	−.442**	[ .710, 2.416]	.643	.030	[1.046, 4.010]	1.030
*Model 2*									
Step χ^2^(5)	304.154*			119.088**			16.207		
Nagelkerke *R*^*2*^	.516			.384			.144		
Age	−.036**	[.940, .990]	.964	−.015	[.953,1.019]	.985	−.130*	[.787, .979]	.878
Sexual attraction	−1.036***	[.300, .421]	.355	−.388***	[.565, .815]	.678	.119	[.709, 1.78]	1.127
Natural	−.261	[.506, 1.173]	.770	−.957**	[ .216, .683]	.384	−.073*	[.353, 2.45]	.930
Discrete	−.648**	[.350, .782]	.523	−2.068***	[ .059, .269]	.126	−1.570	[.053, .816]	.208
Entitativity	−.212	[.511, 1.280]	.809	.206	[.673, 2.242]	1.229	.360	[.520, 3.95]	1.434
Importance	−.168	[.563, 1.270]	.845	1.033***	[1.561,5.058]	2.810	−.656	[.207, 1.29]	.519

^*^p < .05; **p < .01; ***p < .001;

**Table 4:** Sexual orientation beliefs as predictors of sexual identity label (men)

	Gay versus bisexual			Gay versus queer			Bisexual versus pansexual		
Variable	*β*	95% CI	Exp(*β*)	*β*	95% CI Exp(*β*)		*β*	95% CI Exp(*β*)	
*Model 1*									
Step χ^2^(1)	76.39*			7.81*			.283		
Nagelkerke *R*^*2*^	.188			.042			.017		
Age	−.001	[.978, 1.02]	.999	.003	[.969, 1.04]	1.00	−.018	[ .870, 1.11]	.982
Sexual Attraction	−.504**	[ .059, .518]	.604	−.288**	[.626, .899]	.750	−.242	[ .239, 2.58]	.785
*Model 2*									
Step χ^2^(5)	118.11*			48.58*			3.69		
Nagelkerke *R*^*2*^	.284			.170			.217		
Age	.014	[.993, 1.04]	1.01	.022	[ .985, 1.06]	1.02	−.018	[.839, 1.15]	.982
Sexual attraction	−.511***	[.531, .677]	.600	−.227*	[.637, .997]	.797	−.417	[.164, 2.65]	.659
Natural	−1.16***	[.199, .499]	.315	−1.13**	[.165, .639]	.324	1.18	[.165, 63.66]	3.24
Discrete	−.556**	[.382, .860]	.573	−1.61**	[.075, .533]	.200	−1.60	[.004, 9.32]	.203
Entitativity	.545*	[1.01, 2.94]	1.72	−.581	[.215, 1.46]	.559	−.857	[.008, 23.13]	.424
Importance	−.248	[.482, 1.26]	.780	.365	[.619, 3.35]	1.44	−1.20	[.017, 5.39]	.302

^*^p < .05; **p < .01; ***p < .001;

In women, the binomial regression predicting lesbian or bisexual identity found that after controlling for sexual attraction and age, claiming a bisexual identity was predicted by less endorsement of discreteness beliefs (but no other sexual orientation beliefs). That means that with each unit increase in discreteness beliefs, the odds of preferring a lesbian identity increased by 1.91 times (i.e., 1/0.52).

For the binomial regression predicting lesbian versus queer identity, weaker naturalness and discreteness beliefs independently predicted membership in the lesbian category (i.e., with every unit increase in naturalness beliefs the likelihood of a lesbian identity increased by 2.60 times (i.e., 1/0.38), and every unit increase in discreteness beliefs predicted an increase in the likelihood of a lesbian identity by 8.3 times (i.e., 1/0.12). Greater perceived importance predicted queer identity, such that every unit increase in importance was associated by a 2.81-fold increase in the likelihood of a queer identity. Finally, for the binomial regression predicting bisexual versus pansexual identity, weaker naturalness beliefs predicted endorsement of pansexual identity label, such that every unit increase in naturalness beliefs, led to a 1.07-fold increase in the likelihood of a bisexual identity.

In men, the binomial regression predicting gay or bisexual identity demonstrated that weaker naturalness and discreteness beliefs predicted bisexual identity label (such that the likelihood of a gay identity increased by 3.17 for every one unit increase in naturalness beliefs, and 1.74 for every one unit increase in discreteness beliefs). Stronger entitativity beliefs, independently predicted bisexual identity label, such that likelihood of a bisexual identity increased 1.72 times for one unit increase in entitativity beliefs. For the binomial regression predicting gay versus queer identity, weaker naturalness and discreteness beliefs independently predicted endorsement of queer identity label (i.e., such that likelihood of a gay identity increased by 3.08 times for each unit increase in naturalness, and 5 times for each unit increase in discreteness beliefs). Finally, no sexual orientation beliefs predicted bisexual versus pansexual identity membership.

## Discussion

The present study is the first to systematically examine how sexual orientation beliefs differ across a diverse range of sexual identity groups. As predicted, sexual minority men endorsed stronger naturalness and discreteness beliefs than sexual minority women. This is consistent with men’s greater tendency to endorse essentialism generally (Keller, 2005), and perhaps because men’s sexual orientation is more consistent with biological and discrete notions of sexuality (Bailey, 2009), or due to sociological factors, such as greater stigmatization of male same-sex sexuality e.g., leading men with fluid or bisexual attractions to deny such attractions (Herek, 2009). Gay and lesbian individuals reported stronger naturalness and discreteness beliefs, than bisexual, pansexual or queer individuals. Whether this is because homosexuality better conforms to essentialist notions of sexual orientation than other non-heterosexual orientations (Chivers et al., 2004; Rosenthal et al., 2012), or whether this reflects partly a strategic effort to resist heteronormative pressures (Hoyt et al., 2019) is unclear.

Bisexual individuals conceived of sexual orientation in a more discrete manner, and bisexual women specifically viewed sexual orientation as more immutable/biologically based, than individuals adopting the non-traditional labels of queer and pansexual. These findings support the idea that queer and pansexual identity labels are associated with rejection of essentialist beliefs—specifically, a rejection of biological determinism and categorical ways of conceptualizing sexual orientation. To our knowledge, these are the first data suggesting that differences between traditional and non-traditional sexual identity labels are explained by ontological beliefs about sexual orientation in general, rather than by differences in sexual attraction patterns per se.

Among gay and lesbian individuals, those with less-exclusive same-sex attractions reported weaker naturalness, discreteness, and importance sexual orientation beliefs. Arguably, the most straight forward interpretation of these findings is that gay and lesbian individuals with less-categorical sexual attraction patterns adopt beliefs about sexual orientation that are consistent with their non-exclusive same-sex sexual feelings. However, recent evidence suggests that manipulating beliefs about the discreteness and immutability of sexual orientation can actually cause heterosexuals to shift their sexual orientation self-ratings (Morandini et al., 2021). While it is unclear whether this recent experimental finding may generalize beyond heterosexuals, it is possible that a similar effect may have been operating in the sexual minority individuals in the present sample. That is, one potential explanation is that beliefs about sexual orientation may change how one makes sexual orientation self-ratings. Nevertheless, as the present study was cross-sectional in nature, it cannot speak to causal directionality. Further research is needed to clarify the potential bidirectional relationship between sexual orientation beliefs and self-reported sexual attraction patterns.

Contrary to our hypotheses, we did not find evidence suggesting that bisexuals’ sexual orientation beliefs differed as a function of deviance from equally men/women attracted bisexuality, as indicated by nonsignificant quadratic relationships between Kinsey scores and sexual orientation beliefs. This finding is of interest, as a discrete view of sexual orientation—albeit one in which there exist three discrete categories (gay/straight/bisexual), rather than two (gay/straight)—could be expected to be preferred by individuals who view themselves as equally attracted to men and women. However, it seems that discreteness tends to imply a binary perspective, and bisexual individuals (regardless of whether their attraction pattern confirms neatly to the bisexual category (i.e., those equally attracted to men and women) or not (i.e., those leaning toward men or women)) appear to eschew discreteness beliefs. That being said, bisexual men did report stronger discreteness beliefs than bisexual women, as well as stronger entitativity beliefs. Both beliefs are associated with more negative attitudes toward sexual minorities and align with past observations that bisexual men experience higher levels of internalized biphobia than observed in bisexual women (Lea et al., 2014).

Lastly, the present study provides correlational evidence of a relationship between sexual orientation beliefs and the adoption of certain sexual identity labels. Importantly, we found significant effects of sexual orientation beliefs on self-identification even when controlling for Kinsey scores. In other words, the relationships between sexual orientation beliefs and identification were not entirely explained by differences in sexual attraction patterns. For instance, among predominately same-sex attracted individuals, discreteness and naturalness beliefs predicted adoption of a queer identity over a gay identity. Likewise in non-monosexual individuals, weaker naturalness beliefs predicted adoption of a pansexual identity over a bisexual identity (in women, but not men; this null finding in men may be due to the low power to detect an effect given the smallish number of pansexual men in our sample). Finally, controlling for sexual attraction patterns, a greater likelihood to adopt a bisexual identity over a gay identity was predicted by weaker naturalness and discreteness beliefs.

Although correlational, the results of the present study are consistent with the claim that, rather than solely being driven by sexual identity self-label may also be influenced by the beliefs one holds about sexual orientation. This perspective deviates from most accounts of sexual identity development, which tend to imply that distinct sexual identities emerge either from distinct sexual orientations, or from distinct constellations of sexual feelings and experiences (Rieger & Savin-Williams, 2012; Savin-Williams & Vrangalova, 2013). The hypothesis that such sexual identity labeling might, in some instances (e.g., among those adopting non-traditional identity labels), reflect ontological beliefs about sexuality that stem from certain ideological, political, or other cultural influences, is rarely examined. Nevertheless, further research is needed to test the causal claim that sexual orientation beliefs may shape how individuals make sense of their sexual and romantic feelings, and in turn, construct their sexual identity.

### Clinical Implications

These findings have notable implications for clinical work and advocacy with diverse sexual minority communities. First, these findings show that not all sexual minority groups embrace essentialist beliefs about sexual orientation to the same degree. Whereas a “born this way” narrative is widely assumed to be enthusiastically embraced among sexual minorities, the current findings suggest non-monosexuals (and queer and pansexual individuals in particular) are more likely to temper their endorsement of such notions. Therapists working with bisexual, queer, and pansexual individuals should be cautious in assuming that the biological essentialist discourse will be experienced as affirming (Fassinger & Arseneau, 2007), and should consider whether social constructivist ways of discussing their client’s sexuality (e.g., including an emphasis on notions of fluidity, agency, and resistance to heterosexist norms) may fit better with their client’s worldview. Clinicians may also consider how certain sexual identity labels, for instance, queer or pansexual, may reflect more than merely one’s sexual attraction patterns, but also subcultural identification and cultural/political beliefs. Clinicians should also be sensitive to within-group variability of sexual orientation beliefs observed among gay and lesbian individuals. Those whose sexualities are less-exclusive in nature (i.e., show greater other-gender attraction) may adopt continuous and fluid conceptions of sexual orientations as a way of making sense of their own non-prototypical attractions. Again, clinicians should be aware of the ways in which certain manifestations of sexual orientation essentialism may erase experiences of their clients. Lastly, the findings may help guide interventions to reduce stigma. Highlighting the diverse social and cultural factors (including orientation beliefs) which contribute to an individual’s sexual identity may be associated with reduced essentialism and prejudice (e.g., Hegarty, 2010), and increased acceptance of the diversity within sexual minority groups (Bernstein, 2013).

### Limitations

The limitations of the study should be acknowledged to aid in interpretation. First, our study was conducted in a highly educated sample of largely white young adults residing in Australia. Whether these findings can be generalized to populations in non-western cultures is unclear (Henrich et al., 2010), as is whether these findings can even generalize to other western cultures (e.g., North America), and importantly, to ethnic minorities and others poorly represented in our sample. Future research should examine this neglected intersection (Moradi & Grzanka, 2017), to determine whether associations observed in the presented study hold in more ethnically diverse, and less socioeconomically privileged samples.

Next, our method of recruitment (i.e., a convenience sample recruited through LGBTIQ organizations, social media, and university pride) seems to have contributed to our sample being younger, more educated, and more connected LGBTIQ community, in comparison with the LGBTIQ population at large (Ross et al., 2005). Our targeted recruitment toward the LGBTIQ social networks may have also led to an under-representation of mostly heterosexual or heterosexual leaning bisexuals/queer/pansexual individuals who may be less connected to the LGBTIQ online presence, and whose beliefs may not be adequately presented in our sample.

Another limitation of the present study was the small number of queer and pansexual men, which may have resulted in insufficient power, and a failure to detect group differences. The relatively small samples of queer and pansexual participants also precluded us from the confirming the four-factor structure of the Sexual Orientation Beliefs Scale in these populations. Thus, future research should aim to examine whether the current findings replicate among larger samples of queer and pansexual-identified men—and seek to confirm that factor structure of the Sexual Orientation Beliefs Scale in these populations (as well as in diverse samples more generally—particularly given the relatively weak internal reliability of the discreteness subscale for some subgroups in our study). Lastly, the small number of individuals who were not cisgender precluded us from exploring how sexual orientation beliefs are adopted among these populations and how these beliefs may shape their sexual identity. Future research that targets individuals in these populations is needed to explore this question.

A recent body of literature (Grzanka et al., 2016, 2020; Tierney et al., 2021) has used latent profile analysis to examine patterns of responses (among individual participants) on multiple factors of the SOBS. This type of analysis has demonstrated that certain belief factors can have distinct relationships to sexual minority attitudes and identity outcomes, dependent on the context in which they occur—and provide emerging evidence that certain constellations of SOBS are perhaps more informative of sexual minority attitudes than individual factors. The present studies’ approach of examining belief factors in isolation may have failed to identify important constellations of belief that may predict sexual identity group membership, particularly in relation pansexual and queer folk. Future research should aim to examine how profiles of beliefs differ across sexual identity groups.

The present study failed to measure how sexual attraction patterns outside of a unipolar male–female continuum (a measured by the Kinsey Scale) may contribute to “queer” and “pansexual” identification. There is emerging evidence that queer and pansexual identities are not disproportionally adopted by non-cisgender populations (Galupo et al., 2016), but that these labels may be adopted often to signal attractions to non-cisgender bodies or identities (Sprott & Benoit Hadcock, 2018). Future studies may seek to control for attractions to trans-masc, trans-femme, and non-binary bodies/identities, to ascertain if sexual orientation beliefs predict pansexual and queer identities above and beyond these potentially identity shaping attractions.

### Conclusion

The present study is the first to examine differences in ontological beliefs about sexual orientation between diverse sexual identity groups. Gay and lesbian individuals were most likely to embrace belief sets that were consistent with a biological determinist view of sexual orientation (i.e., increased naturalness and discreteness beliefs). Bisexual men and women both demonstrated lower endorsement of naturalness and discreteness beliefs. Further, those adopting non-traditional sexual identity labels (i.e., pansexual and queer) were most likely to eschew essentialist sexual orientation beliefs. Interestingly, we also found that sexual orientation beliefs predicted the adoption of specific identity labels even when controlling for sexual attraction patterns. These findings indicate that sexual orientation beliefs predict an individual’s choice of their sexual identity label. However, the cross-sectional nature of the study does not allow for causality in this relationship to be inferred. Regarding clinical implications, the current findings suggest the need for sensitivity to possible differences in sexual orientation beliefs as a function of the client’s sexual identity label.

## Data Availability

Data are included as electronic supplementary material

## Footnotes

1 Two items (17 and 18) were dropped from the Discreteness subscale for all participants (in all sexual identity groups) to ensure that Cronbach’s alpha was above. 60 in pansexual men and women.
